# Generation of Hepatobiliary Cell Lineages from Human Induced Pluripotent Stem Cells: Applications in Disease Modeling and Drug Screening

**DOI:** 10.3390/ijms22158227

**Published:** 2021-07-30

**Authors:** Mattia Pasqua, Roberto Di Gesù, Cinzia Maria Chinnici, Pier Giulio Conaldi, Maria Giovanna Francipane

**Affiliations:** 1 Fondazione Ri.MED, 90133 Palermo, Italy; mpasqua@fondazionerimed.com (M.P.); rdigesu@fondazionerimed.com (R.D.G.); cchinnici@fondazionerimed.com (C.M.C.); 2 Dipartimento della Ricerca, IRCCS ISMETT, 90127 Palermo, Italy; pgconaldi@ismett.edu; 3 McGowan Institute for Regenerative Medicine, University of Pittsburgh, Pittsburgh, PA 15219, USA

**Keywords:** iPSCs, hepatocytes, cholangiocytes, disease modeling, drug testing, tissue engineering

## Abstract

The possibility to reproduce key tissue functions in vitro from induced pluripotent stem cells (iPSCs) is offering an incredible opportunity to gain better insight into biological mechanisms underlying development and disease, and a tool for the rapid screening of drug candidates. This review attempts to summarize recent strategies for specification of iPSCs towards hepatobiliary lineages —hepatocytes and cholangiocytes—and their use as platforms for disease modeling and drug testing. The application of different tissue-engineering methods to promote accurate and reliable readouts is discussed. Space is given to open questions, including to what extent these novel systems can be informative. Potential pathways for improvement are finally suggested.

## 1. Introduction

The hepatobiliary system, which includes the liver and the biliary tract, is crucial for several physiological processes. In a simplistic view, the liver plays a central role in metabolic control, drug detoxification, and protein synthesis, while the biliary tract modifies and releases the bile produced in the liver facilitating intestinal lipid digestion and absorption [1].

The human liver is classically divided into left, right, caudate and quadrate lobes. On the histological level, the lobule represents the liver functional unit, which receives blood through branches of the portal vein and hepatic artery. On the other hand, the biliary tract consists of a network of ducts located inside or outside the liver, referred to as intrahepatic bile ducts (IHBDs) and extrahepatic bile ducts (EHBDs), respectively. The IHBDs can be further classified in intrahepatic large and small bile ducts, whereas the EHBDs consist of left and right hepatic ducts, cystic duct, common bile duct, and gallbladder [2].

Microscopically, two main types of epithelial cells compose the hepatobiliary system: the hepatocytes, which are the most abundant cells in the liver parenchyma, and the biliary cells or cholangiocytes, which line both IHBDs and EHBDs. Hepatocytes and intrahepatic cholangiocytes are derived from the hepatoblast [3]. During development, those hepatoblasts adjacent to portal veins will form the ductal plate and become cholangiocytes. Notch signaling in both hepatoblasts and cells of the portal mesenchyme is crucial for this step of cholangiocyte specification [4]. By contrast, hepatoblasts farther away from the portal mesenchyme will differentiate into mature hepatocytes [4]. The origin and development of extrahepatic cholangiocytes is markedly different. In fact, these cells share a common developmental origin with the ventral pancreas [5,6].

Under homeostatic conditions, both liver and biliary tissues are maintained by simple proliferation of existing hepatocytes and cholangiocytes [7]. Tissue recovery after injury can occur through either a similar mechanism or through cellular reprogramming. In experimental and clinical biliary tract injury, and in various types of liver injury, cells with mixed hepatobiliary phenotype arise along the canals of Hering (the anatomic interface between cholangiocytes and hepatocytes), the portal bile duct, and the periportal areas [8,9,10,11]. These hybrid and proliferating cells were variably termed oval cells (in rodents), ductular reactions, and liver progenitor cells, among others [12,13,14,15]. The liver progenitor cell designation implies an origin from tissue-specific stem cells, however, there is no evidence to suggest that tissue-specific stem cells exist in the adult liver as in intestine and skin. Lineage tracing, gene expression analyses and mouse genetics rather suggest that the cells with mixed hepatobiliary phenotype, which occur during disease, might derive from the reprogramming of adult hepatocytes and cholangiocytes [16,17,18,19,20]. In other words, hepatocytes and cholangiocytes would function as ‘facultative’ stem cells for each other when conventional repair mechanisms are impaired. The potential of hepatocytes to rescue intrahepatic cholangiocytes—phenomenon referred to as hepatocyte transdifferentiation—is well accepted and has been recently supported by additional evidence [21], while the opposite has been highly debated [22,23]. Recent investigations in animal models of liver injury and regeneration, however, support this idea [24,25]: cholangiocytes are not cinderellas to the hepatic regenerative response [26], but can be the providers of new hepatocytes when surviving hepatocytes are compromised in their ability to proliferate.

Differently from the liver, peribiliary glands around the bile ducts possess multipotent stem cells, which self-renew and can differentiate into hepatocytes, cholangiocytes or pancreatic islets, depending on the microenvironment [27]. Similar cells—presumably committed progenitor cells—are found in gallbladder’s mucosal crypts [28,29]. These discoveries have significant clinical implications. A wide variety of diseases and conditions affect the liver and the biliary system, and many patients, unfortunately, progress into chronic conditions, which require liver transplantation. Liver transplantation itself might cause biliary complications in transplant recipients [30]. In vitro expansion of native hepatocytes, cholangiocytes, liver progenitor cells, and/or multipotent stem/progenitor biliary cells could theoretically provide key components for the development of screening platforms to identify candidate treatments and block disease progression, and/or building blocks for liver and biliary tissue engineering. Barriers to the translation of liver and biliary primary cells into research and clinical applications, however, include the difficult task of cell isolation and purification, aggravated by the chronic shortage of suitable donor organs and tissues, as well as the lack of robust protocols for long-term maintenance of cell function. Cell lines possess numerous advantages with respect to primary cells, including an almost unlimited proliferative capacity, a relatively cheap culture process, and reproducibility of metabolic performance across different batches (one of the main pitfalls of using primary cells). Nonetheless, liver cell lines must still demonstrate a mature state and metabolic functions comparable to human primary cells as well as being safe and non-tumorigenic [31]. As such, much effort has been devoted in recent years to the search for alternative sources. One such source is mesenchymal stromal cells (MSCs). MSCs of different origin are more easily isolated and propagated than liver primary cells [32]. A plethora of studies has established that MSCs can differentiate in vitro into multiple lineages, although satisfactory results were obtained only in the case of MSC conversion into mesodermal lineages (osteocytes, chondrocytes, and adipocytes) [33]. Yet, MSCs remain a promising source for liver repair thanks to their well-documented paracrine activities [34,35]. More recently, the development of the induced pluripotent stem cell (iPSC) technology, which allows to convert one cell type into another [36], has created a potentially inexhaustible supply of cells for large-scale toxicity testing and drug screening, and an outstanding source of patient-specific cells for disease modeling. Both hepatocytes and cholangiocytes have been generated from iPSCs [37]. How closely such iPSC-derived cells can recapitulate in vivo cell functions is subject of intense investigation.

In this review, we briefly describe attempts of hepatocyte and cholangiocyte generation from human iPSCs, and discuss their application in disease modeling and drug testing. Finally, we discuss the importance of adopting three-dimensional (3D) cell culture systems to build functional tissue surrogates. We review the current literature around the use of tissue-engineering approaches to improve iPSC specification and maturation towards hepatobiliary lineages.

## 2. Generation of Hepatocytes and Cholangiocytes from Human Induced Pluripotent Stem Cells and Their Applications

### 2.1. Introduction to iPSC Technology and Lessons from Development for iPSC Differentiation into the Hepatoblast

The past decades have seen remarkable progress in the development and use of human cell-based models to study organ development as well as mechanisms underlying disease. With the discovery that many tissues and organs contain a small number of adult stem cells, researchers began to focus on establishing long-term in vitro 3D culture systems that could recapitulate the complex cellular diversity and function of native organs [38,39]. However, challenges in the identification, isolation, and expansion of many adult stem cell populations, as well as concerns regarding the potency of stem cells derived from a diseased or injured organ created a need for the development of novel and renewable cell sources for basic research and potential clinical applications. The turning point occurred in 2006, when Yamanaka and Takahashi discovered that it is possible to convert somatic cells into cells with pluripotent features [36]. Their reprogramming strategy was based on retroviral transduction of four transcription factors, namely octamer-binding transcription factor 3/4 (Oct3/4), sex-determining region Y-box 2 (Sox2), krupper-like factor 4 (Klf4), and cellular myelocytomatosis (c-Myc). Since this protocol was reported, non-integrating strategies, including but not limited to those based on the use of episomal vectors or Sendai virus, were developed to prevent the potentially deleterious effects of random and stable integration of the four transgenes into the genome of infected cells [40]. In parallel, cell-adhesive matrices (including but not limited to vitronectin and fibronectin) progressively replaced traditionally used feeder layers, to facilitate cell preparation and also clinical application of the iPSC derivatives [41,42]. Current effort is directed towards the identification of small molecules that can increase reprogramming efficiency and quality, or even replace the transcription factors [43] and the multitude of recombinant growth factors, which are used in virtually all directed differentiation protocols [44,45]. Small molecules are more stable and cost-effective than recombinant growth factors, and their use would be convenient for large-scale cell production. Yet, significant obstacles to clinical translation exist. iPSCs display cancer stem cell features, and still there exists no differentiation protocol that can guarantee the generation of a cellular product which is devoid of contaminating undifferentiated or partially differentiated iPSCs [46,47]. These cells could potentially lead to a malignant growth in the patient. As such, iPSC derivatives lend themselves better for in vitro disease modeling and drug/toxicity testing at this time, although they might one day provide unprecedented opportunities to rebuild functional organs.

Both hepatocyte- and cholangiocyte-like cells can be obtained from iPSCs using step-wise protocols that mimic embryonic development [3]. Both protocols share the induction of a definitive endoderm (DE) state followed by DE differentiation into a bipotential progenitor, the hepatoblast [48,49].

In vertebrates, the transforming growth factor beta (TGF-β)-related ligand Nodal is indispensable for the formation of the DE [50,51,52]. As such, a related ligand, Activin A, is commonly used to induce DE in pluripotent stem cells. While high doses of Activin A are sufficient to induce DE in vitro in both embryonic stem cells (ESCs) and iPSCs, this process is more efficient in low serum culture conditions [53] or when phosphatidylinositol 3-kinase (PI3K) signaling is suppressed [54]. For this reason, PI3K pathway inhibitors such as LY294002 are often added to the culture medium to induce DE. Of note, in vivo, nodal signals act in concert with other growth factors belonging to the fibroblast growth factor (FGF), wingless-type MMTV integration site (Wnt) and bone morphogenetic protein (BMP) families [55]. This finding has led to the inclusion of additional signaling molecules such as FGF2, Wnt-3a, the glycogen synthase kinase (GSK-3) inhibitor/Wnt agonist CHIR99021, and/or BMP4 in the DE induction medium [56,57,58]. FGF2 and BMP4 are also often used to direct hepatic fate-specification from DE, and also dimethyl sulfoxide (DMSO) has been used to the same purpose. However, while DMSO successfully induced hepatoblasts from ESC-derived DE [59], its ability to induce the same cells from iPSC-derived DE remains controversial. Indeed, DMSO alone induced differentiation of iPSC-derived DE toward hepatoblasts in a study [44], while in a different report, caused obvious toxicity to the cells and was not sufficient to induce hepatic differentiation [45].

Recently, a search for cost-effective protocols to support hepatoblast expansion with preserved bipotency has led to the establishment of a chemically defined culture medium consisting of a mixture of the TGF-β inhibitor A83-01, CHIR99021, the hepatocyte growth factor (HGF) receptor agonist N-hexanoic-Tyr-Ile-(6) amino hexanoic amide (Dihexa), the protein kinase A (PKA) signaling activator Forskolin (FSK), sonic hedgehog agonist (SAG), and vitamin C [60]. Small molecules, however, present some hidden risks. Indeed, the pathways which they modulate are regulated by various feed-forward and feed-back loops and cross-talks. Therefore, while notions from developmental biology indicate that differentiation into a given cell lineage is dependent upon a specific sequence of cellular events, it is very challenging to control these events experimentally, and this is aggravated by a lack of proper knowledge of the specific properties of most small molecules currently in use.

Regardless of how they were obtained, established hepatoblasts are later treated with specific growth factors and modulators of key developmental signaling pathways in order to trigger differentiation into hepatocytes or cholangiocytes. Among determinants of hepatoblast cell fate is the Notch signaling pathway. Notch activation in hepatoblasts results in repression of hepatocytic differentiation and induction of biliary differentiation [4]. In physiological conditions, it is the interaction of Notch2 expressed in the hepatoblasts with the JAGGED1 (JAG1) ligand expressed in the developing portal mesenchyme that drives hepatoblast differentiation into cholangiocytes [4]. Disruption of IHBD development in mice following liver-specific inactivation of *Notch2*, but not of *Notch1*, indicated that Notch2 is the dominant receptor responsible for the establishment of a functional biliary system [61]. On the other hand, the critical role of JAG1 was supported by the finding that mutations in *JAG1* result in humans in a condition called Alagille syndrome, which is characterized by a paucity of IHBDs, as well as abnormalities in other organs [62]. Notch signaling controls the expression of liver-enriched transcription factors, including members of the hepatocyte nuclear factor (HNF) and the CCAAT/enhancer binding protein (C/EBP) families [4]. HNF1α, HNF4, and C/EBPα are crucial for the development of hepatocytes, whereas HNF1β and HNF6 are essential for the development of the biliary lineage [4].

The Notch signaling is also important for normal development of EHBDs. Indeed, deletion of the Notch target gene *Hes1* in mice results in gallbladder agenesis and severe hypoplasia of EHBDs [63]. Interestingly, biliary epithelium in *Hes1*^−/−^ mice ectopically expresses the proendocrine gene *Neurog3*, differentiates into endocrine and exocrine cells, and forms acini and islet-like structures in the mutant bile ducts [63]. Thus, HES1 determines biliary organogenesis by preventing the pancreatic differentiation program.

The importance of Notch signaling in cholangiocyte specification from iPSCs was also confirmed by inhibiting the formation of the Notch intracellular domain (NICD) with the use of the gamma-secretase (GSI) inhibitor L-685,458, which suppressed the expression of JAG1, HES1, and NOTCH2 and hence, cholangiocyte organoid formation [64].

DE induction in iPSCs and subsequent hepatic specification can be monitored through analysis of specific markers, including but not limited to SOX17 for DE, and HNF4α, alpha-fetoprotein (AFP), and T-Box Transcription Factor 3 (TBX3) for hepatoblasts. In the following paragraphs, we will dive into the most common protocols of hepatoblast differentiation into hepatocyte- or cholangiocyte-like cells, and provide some examples of how these cells can be exploited for disease modeling and drug testing.

### 2.2. Generation of Hepatocyte-Like Cells and Applications in Disease Modeling and Drug Testing

Since the first reports of hepatocyte-like cell generation from iPSCs [48,65], there has been a growing interest in developing increasingly efficient and reproducible protocols for their obtainment. Hepatic induction protocols often use FGF2 and BMP4 for hepatoblast differentiation, and HGF, oncostatin M (OSM), and dexamethasone (DEX) for hepatic maturation [66]. Many of the characteristics of native hepatocytes are recapitulated in hepatocyte-like cells obtained following these protocols. Among these are liver-specific protein expression (such as albumin [ALB], asialoglycoprotein receptor [ASGPR], alpha-1 antitrypsin [AAT]), glycogen and lipid storage, urea synthesis, uptake of low-density lipoprotein, metabolism of indocyanine green (ICG), and binucleated morphology [65,67]. Moreover, induced hepatocytes maintain functional activity of major cytochrome P450 (CYP) isoforms and possess active efflux capacity of marker substrates (such as the multidrug resistance-associated protein 2 substrate CDFDA) into bile canalicular compartments [68]. Of note, induced hepatocytes are able to respond to hepatotoxins, such as acetaminophen, troglitazone, nefazodone, and theophylline [65,68].

With the aim of discovering some promising chemical compounds to replace the use of growth factors (including HGF and OSM) in the last stage of hepatocyte differentiation, Siller et al. established a protocol to induce hepatic progenitor cells from DE based on the use of small molecules DMSO, DEX, the glucocorticoid receptor agonist hydrocortisone-21-hemisuccinate (HC), and Dihexa [44]. The differentiated cells demonstrated similar levels of function (metabolic potential, production of serum proteins, ability to store glycogen, and uptake of ICG) with respect to hepatic progenitor cells derived using growth factor-based approaches [44]. Similarly, Du et al. established a protocol to induce hepatic progenitor cells from DE based on the use of A83-01, along with the histone deacetylase (HDAC) inhibitor sodium butyrate and DMSO [45]. Such small molecule-based differentiation protocol proved to be more efficient than growth factor-driven protocol. Moreover, expression of hepatic markers and liver-specific activities could also be maintained [45].

Xenobiotic metabolism, involving CYP enzyme family among others, plays an important role in drug metabolism and biotransformation of endogenous and exogenous compounds. Therefore, the hepatocytes derived from iPSCs must express those enzymes if intended for drug testing. Among major CYP isoenzymes in the adult liver are CYP1A2 and CYP3A4. Activity of CYP3A4 is of particular importance, as this isoenzyme is responsible for the metabolism of the majority of drugs [69]. CYP activities in induced hepatocytes were stable for at least one week in culture, while they dramatically decreased in human primary hepatocytes during the first 48 h of culture [70]. However, low CYP1A2 and CYP3A4 expression levels in induced hepatocytes suggested they had an immature phenotype, although the concomitant low levels of the fetal isoenzyme CYP3A7 indicated that they no longer had a fetal phenotype [70]. Similar data were obtained by Medine et al., who established induced hepatocytes with ~34% CYP3A and ~1% CYP1A2 activity of primary human hepatocytes [71]. Importantly, Holmgren et al. demonstrated that induced hepatocytes could be maintained for at least 2 weeks in culture, thus enabling a repeated-dose chronic exposure to hepatotoxic compounds [72]. Even more striking were results from Khetani’s group, who showed that induced hepatocytes from multiple donors display significantly greater levels and stability of major liver functions (i.e., CYP450 activities) for ~4 weeks [73], when they are micropatterned onto collagen-coated domains of empirically optimized dimensions, surrounded by 3T3-J2 murine embryonic fibroblasts, and then sandwiched with a thin layer of Matrigel [74]. This platform improved the sensitivity of drug toxicity detection over conventional cultures. It correctly classified 24 of 37 hepatotoxic drugs, while conventional confluent cultures failed to detect several.

While genome-wide array analysis has recently confirmed that induced hepatocytes show signs of immaturity [75], evidence continues to accumulate in support of induced hepatocytes for hepatotoxicity studies, not only over primary hepatocytes, but also over most hepatoma cell lines [76]. To demonstrate the utility of induced hepatocytes as a platform for drug screening, Choi et al. established induced hepatocytes from patients with AAT deficiency [76], for which there is currently no drug or gene therapy available. Through a blind large-scale drug screening, they identified five clinical drugs able to reduce AAT accumulation in the induced cells. Similarly, Cayo et al. generated induced hepatocytes from patients with homozygous familial hypercholesterolemia (HoFH) and screened a library of 2320 existing drugs [77]. Authors looked for compounds that could reproducibly decrease apolipoprotein B secretion, and identified several cardiac glycosides that could meet this challenge. Gurevich et al. instead, generated induced hepatocytes from non-alcoholic steatohepatitis (NASH) donors [78]. The NASH induced hepatocytes displayed increased lipid accumulation in response to fatty acid (FA) exposure, and interestingly, exhibited spontaneous lipid accumulation in the absence of FA supplementation, thus mimicking a feature of in vivo NASH hepatocytes. Of note, gene expression signatures similar to those of liver tissues from patients with NASH could be also obtained in iPSC-derived hepatic organoids from healthy control patients following incubation with free FA [79]. The bile canaliculi network in these organoids was disrupted, and a mislocalization of dipeptidyl peptidase IV (DPPIV), could be observed, which is indicative of loss of polarity within the hepatocytes.

Induced hepatocytes were also established from urine cell-derived iPSCs, taken from patients with Wilson disease and MEDNIK syndrome (mental retardation, enteropathy, deafness, peripheral neuropathy, ichthyosis, keratodermia), which are two inherited copper (Cu) metabolism-related liver disorders caused by mutations in *ATP7B* and *AP1S1* genes, respectively [80]. *ATP7B*, encodes for the copper-transporting ATPase ATP7B, while *AP1S1*, encodes for the σ1A subunit of adaptor protein complex 1 (AP-1), which directs intracellular trafficking of the ATP7B protein. ATP7B normally resides in the trans-Golgi network (TGN). However, when intracellular Cu^2+^ levels become too high, ATP7B translocates to the bile canaliculus, where excess Cu^2+^ is excreted. Interestingly, investigation of induced mutant hepatocytes revealed unexpected and new trafficking phenotypes of ATP7B. Indeed, the Wilson disease-causing ATP7B-H1069Q mutation *per se* did not preclude trafficking of ATP7B to the TGN. Instead, it prevented its Cu^2+^-induced polarized redistribution to the bile canalicular domain, which could not be reversed by the pharmacological folding chaperones curcumin and 4-phenylbutyrate. On the other hand, MEDNIK syndrome-causing *AP1S1* mutations were not associated with defects in the Cu^2+^-induced redistribution of ATP7B to the bile canaliculi. Overall, this study demonstrated the importance of investigating the response of endogenously expressed mutant proteins to novel therapeutic strategies in patients’ own cells.

A combination of human iPSCs with genetic correction can accelerate the generation of clinically relevant cells for basic research and even autologous cell-based therapies. Footprint-free gene correction of AAT was achieved in iPSCs derived from an AAT-deficient patient with the Z mutation (Glu342Lys), using ZFNs and piggyBac DNA transposon [81]. The transcription activator-like effector nuclease (TALEN) technology was used by Choi et al. for the same purpose [82]. Alongside the above-mentioned genome editing systems, the clustered-regularly-interspaced-short-palindromic-repeats/Cas-associated 9 (CRISPR/Cas9) system and its newest evolution base-editing are making gene editing faster, cheaper, and even more efficient. CRISPR/Cas9 was used to permanently correct a 3-base pair homozygous deletion in exon 4 of the low-density lipoprotein receptor (LDLR) of patient-derived HoFH iPSCs [83]. The genetic correction restored LDLR-mediated endocytosis in induced HoFH hepatocytes, demonstrating that CRISPR-mediated genome editing can be successfully used to normalize HoFH cholesterol metabolism deficiency at the cellular level.

Not only as an in vitro platform for metabolic disease modeling and drug discovery, but also induced hepatocytes were exploited to study viral infections, including those from hepatitis C virus (HCV) [84,85] and hepatitis B virus (HBV) [86], and test antiviral drugs. Pluripotent stem cells (both ESCs and iPSCs) and DE were not permissive to HCV infection, whereas induced hepatocytes could be persistently infected, and secreted infectious particles in the culture medium [84]. Transition to HCV permissiveness during the in vitro differentiation process seemed to require both the activation of positive factors (microRNA 122 [miR122], epidermal growth factor receptor/ephrin type-A receptor 2 [EGFR/EphA2], phosphatidylinositol 4-kinase III α [PI4KIIIα] etc.) and the downregulation of antiviral genes such as *IFITM1*, which encodes the interferon induced transmembrane protein 1 [84]. Induced hepatocytes supported the entire life cycle of HCV, including inflammatory responses to infection [85]. Furthermore, induced hepatocytes could support long-term infection of multiple HCV genotypes in vivo, following engraftment and further maturation in mouse livers [87]. Similarly, fully differentiated induced hepatocytes, but not cells at earlier stages of differentiation, supported productive HBV infection [86]. Infection could be maintained over a period of weeks by inhibiting the innate immune response with inhibitors of the Janus kinase (JAK) family or the signaling intermediate TANK binding kinase 1 (TBK1) [86]. Conversely, treatment with the HBV reverse-transcriptase inhibitor entecavir, or an alternate antiviral, interferon beta (IFN-β) abrogated the infection [86]. To further improve the hepatic maturation and facilitate HBV infection, a trans-well co-culture system of induced hepatocytes with non-parenchymal cells was very recently established [88]. Liver sinusoidal endothelial cells (LSECs) enhanced HBV infection through a paracrine mechanism [88]. Specifically, LSECs secreted epidermal growth factor (EGF), and EGF, in turn, modulated HBV infection dose-dependently via EGFR-mediated endocytosis pathways [88].

Overall, the reviewed studies provide a strong evidence of the utility of iPSC-derived hepatocytes for modeling both genetic and viral diseases, and for drug repurposing screening strategies, although there is still much room for improvement.

### 2.3. Generation of Cholangiocyte-Like Cells and Applications in Disease Modeling and Drug Testing

Knowledge of the mechanisms driving bile duct development in the mouse guided the design of protocols for cholangiocyte generation from iPSCs. Previously, we described how Notch pathway functions to specify the cholangiocyte lineage from the hepatoblast. The TGF-β1, EGF, and HGF pathways were identified as key determinants promoting further maturation of the cholangiocyte-committed cells [89,90].

To our knowledge, the first report about the possibility of generating cholangiocyte-like cells from iPSCs appeared in 2014. Dianat et al. grew hepatoblasts for three days in growth hormone (GH) and EGF, and then, for additional three days, in interleukin (IL)-6 [49]. Cells were therefore transferred onto collagen I-treated wells, differentiated for three days in IL-6, and then, for two additional days, in taurocholate bile salt. In the course of differentiation, cells progressively acquired a cuboidal morphology (typical of small cholangiocytes) and expression of cholangiocyte markers. Induced cholangiocytes expressed cytokeratin (CK) 7, CK19, HNF6, SOX9, and cystic fibrosis transmembrane conductance regulator (CFTR) at the protein level and showed primary cilia. To assess the potential of induced cholangiocytes to form cysts and tubules, a 3D culture system was used. Through this system, induced cholangiocytes demonstrated the ability to form polarized cysts that could transport the fluorescent bile acid cholyl-L-lysyl-fluorescein (CLF). Luminal extrusion of bile acids is mediated by the apical Na^+^-dependent bile acid transporter (ABAT), and is a feature of differentiated cholangiocytes. In another study, a combination of Activin-A, retinoic acid, and FGF10 were used to differentiate hepatoblasts into cholangiocyte progenitors (CP) [64]. To promote maturation of CPs, 3D culture conditions were used. CPs grown in 3D proliferated rapidly, organized into ring-like structures after 48-72 h and within 5–7 days gave rise to cystic organoids and branching tubular structures bearing primary cilia. The organoids expressed biliary markers, including CK7, CK18, CK19, HNF1β, gamma-glutamyltransferase (GGT), JAG1, NOTCH2, CTFR, secretin receptor (SCR), somatostatin receptor 2 (SSTR2), aquaporin 1 (AQP1), and anion exchange protein 2 (AE2) at levels similar to those in primary cholangiocytes. The induced cholangiocytes displayed a range of functions of the native biliary epithelium, corresponding however to intrahepatic cholangiocytes with fetal characteristics [64]. The multidrug resistance protein 1 (MDR1) fluorescent substrate rhodamine 123 was detected in the lumen of organoids derived from induced cholangiocytes, confirming MDR1 functionality. Active export of CLF from organoid lumen was also demonstrated. The same protocol was later followed to generate cholangiocyte-like cells from skin fibroblasts of a patient homozygous for the most common cystic fibrosis (CF) mutation ΔF508. Wild type cholangiocyte-like cell organoids appropriately modified intracellular chloride in response to media with varying concentrations, whereas no change was observed in CF-cholangiocyte-like cells, confirming the absence of functional CFTR in these cells. Incubation of CF cholangiocyte-like cells with the CFTR corrector VX809 for 48 h increased CFTR function. This effect was negated by CFTR inhibitor 172, confirming that the phenotypic rescue of CF-cholangiocyte-like cells by VX809 was dependent on improved CFTR function. Thus, mature, functional cholangiocytes can be derived from human iPSCs and utilized to model biliary diseases in vitro. More recently, this protocol was used by Takeishi et al. with some modifications [91]. To differentiate hepatoblasts into CPs, cells were exposed to Activin A, FGF10, and retinoic acid for four days. Markers of early biliary specification including *SOX9*, *HNF1β*, and *CFTR* were upregulated at the mRNA level, and *CK7* and *AFP* were also detected, indicating a transition into CP. To enhance natural bile duct maturation, authors added a differentiation stage based on the role of Notch and TGF-β signaling, as well as signaling through IL-6 and EGF. Specifically, they added EGF, IL-6, DEX, sodium pyruvate, TGF-β1, and soluble Delta-like protein 1 (DLL-1, a ligand for Notch receptors). These factors induced expression of the mature cholangiocyte markers SOX9, CK19, and CK7, and in most cells eliminated AFP expression. Importantly, CFTR and Inositol 1,4,5-Trisphosphate Receptor Type 3 (ITPR3) were induced. Next, the functionality of the induced cholangiocytes was characterized in 3D culture conditions through organoid formation. Organoids of induced cholangiocytes were able to actively export CLF from their lumen.

To induce cholangiocyte maturation from hepatic progenitors, another group exposed cells daily to TGF-β [92]. Induced cholangiocytes lacked significant expression of early hepatic markers and showed instead high expression of multiple cholangiocyte markers including *CK19*, *CK7*, *CFTR*, *PKD2* (Polycystin 2), and *AE2* by RT-PCR, and biliary proteins by western blot including acetylated α-tubulin, AQP1, ABAT, and SSTR2, along with CK7 and CK19. RNA-seq analysis next indicated that induced cholangiocytes had an intermediate molecular phenotype between the freshly isolated intrahepatic cholangiocytes and the cholangiocyte cell lines. Induced cholangiocytes formed primary cilia following serum starvation and had intact calcium signaling. Moreover, they formed duct-like structures in a 3D collagen/Matrigel culture system. Finally, retrograde infusion of induced cholangiocytes in the mouse revealed engraftment capability.

A co-culture with OP9 stromal cells, which are known to express different Notch ligands including JAG1, was used by other authors to induce maturation of cholangiocytes from hepatoblasts [93]. When co-cultured on OP9 cells for 9 d, hepatoblasts formed distinct clusters of CK19+ cells that downregulated ALB expression, suggesting that they had undergone the initial stage of cholangiocyte specification. Co-culture of hepatoblasts with OP9 cells in the presence of EGF, HGF and TGF-β1 further promoted the development of a population that expressed markers of more mature cholangiocyte including CFTR, ABAT and α-tubulin. Expression of Notch targets HES1, HES5 and HEY1 was upregulated after 9 d of culture on OP9 cells in the presence of EGF, HGF and TGF-β1. GSI blocked downregulation of ALB, reduced the proportion of CK19+ cells and inhibited the development of branched structures in the cultures, indicating that these effects were mediated by Notch signaling. Chimeric aggregates consisting of day-25 hepatoblasts and OP9 stromal cells were later cultured in gels made of collagen type-1 and Matrigel in the presence of HGF, EGF, and TGF-β1. Within 2 weeks of culture, the aggregates gave rise to 3D structures that displayed either a tubular and ductal morphology, a hollow cyst morphology or a mixture of both. The generation of cysts and duct structures was dependent on Notch signaling, as these structures did not develop in the presence of GSI. Induced cholangiocytes acquired apico-basal polarity, a feature of mature epithelial ducts, and formed ductal structures ectopically in vivo, after transplantation in a Matrigel plug into mammary fat pads of nonobese diabetic–severe combined immunodeficient–interleukin 2rγ knockout (NOD-SCID-*IL2rγ*^−/−^; NSG) mice. The activity of MDR1 and CFTR was next evaluated by using rhodamine 123 and FSK. Finally, biliary CF was modeled in vitro and the efficacy of potential therapeutic agents (chemical correctors VX-809/Corr-4a) was demonstrated using a cyst-swelling assay.

Thus, similar to induced hepatocytes, induced cholangiocytes might set the foundation for the development of high-throughput drug screening platforms for multiple diseases in the future using patient-derived cells. What emerges from the literature is that while it is possible to differentiate iPSCs towards hepatocyte-like cells with the only use of small molecules modulating crucial signaling pathways, cholangiocyte protocols still suffer from the dependence of growth factors, interleukins, and other compounds for both specification and maturation stages. Table 1 summarizes commonly used small molecules for the generation of both hepatobiliary lineages. Information is given about which pathway each molecule modulates, and the specific effect on the pathway or the cells.

## 3. Toward Improved Human iPSC-Based Hepatobiliary Models

Liver cells generated from iPSCs hold great promise for accelerating pharmaceutical testing and drug development without the use of animals. How much culture conditions for iPSCs closely mimic native hepatic/biliary niches and to which extent the iPSC-derived cells resemble their native counterparts are key factors to consider when using these cells for drug testing. A widespread concern exists about the use of two-dimensional (2D) cell culture systems for the generation of iPSCs and their subsequent differentiation. This fast and cost-effective approach is based on maintaining cells as monolayers on flat plastic surfaces, which can be pre-treated with different coating materials to promote cell adhesion and growth. The advent of increasingly sophisticated cell analysis tools, however, has shed light on important limitations associated with such cell culture systems. It is now well accepted that cells in a 2D monolayer often assume unnatural conformations, which might result in unrealistic biological responses. For example, cells grown in vitro onto rigid and flat surfaces (e.g., culture flasks, multi-well plates) often undergo apical-basal polarization, which is unnatural for cytotypes such as MSCs [94]. This has spurred an interest over the last 20 years in developing cell culture systems that could allow cells to spatially organize into more realistic structures [94]. Physiologically, cells are embedded into the extracellular matrix (ECM), which provides structural support as well as dynamic signaling cues that influence cell fate [94]. This consideration has guided the development of biomaterials that could provide specific biological and mechanical cues to the differentiating cells in vitro. The use of such biomaterials has allowed 3D cell culture technology to rapidly evolve from cell aggregate-based methods that recapitulate cell–cell interactions to more complex scaffold-based methods that also recapitulate cell-to-ECM interactions [95]. Among these methods, the matrix-assisted 3D culture system seems to be one of the most promising and versatile strategies, with cells embedded within an ECM-like matrix that supports their growth and promotes the deposition of newly produced ECM. The ECM-like matrix also protects the cells from external degradative stimuli, such as a host’s immune system response after implantation in vivo [96].

Hydrogels, which are a class of water-retaining materials with great ECM similarity, represent candidate biomaterials for the fabrication of ECM-like matrix [97]. Chemically, hydrogels are polymeric compounds composed of a main chain (backbone) that can be used as an anchorage point for several bioactive molecules or secondary polymer molecules (side chains) characterized by different chemical–physical properties. Hydrogels are classified as natural, if polymers derive from a natural source (e.g., collagen, fibrin, hyaluronic acid), or synthetic/semi-synthetic, if they derive, completely or partially, from chemical reactions [98]. Such chemical heterogeneity enables a wide range of structural modifications, and thus modulation of biological responses from embedded cells. For example, matrices made of hydrogels with inert structure (e.g., alginates) limit cell-matrix interactions, promoting the formation of self-assembling 3D structures such as spheroids. Hepatocyte aggregates obtained using this approach showed improved biological activity in vitro, including metabolic and biotransformation capabilities [99,100,101]. Conversely, the insertion of bioactive peptides into the polymeric backbone allows cell-matrix interactions, promoting cell adhesion while preventing cell dedifferentiation. This approach was followed by Ye et al. [102], who used an ECM-like hydrogel matrix made of polyisocyanopeptides (PIC) and functionalized with laminin-111, as a scaffold for human hepatocytes. Matrix functionalization allowed to maintain hepatic phenotypes in cultured cells and to develop a human liver organoid in vitro. In addition, MacPherson et al. developed a nanofibrous 3D hydrogel functionalized with the integrin-binding motif Arg-Gly-Asp (RGD) and with tailored mechanical properties, to recreate an in vitro environment suitable for maintenance of primary hepatocytes. This scaffold promoted hepatocyte survival, hepatic functions and expression of hepatocyte-specific genes [103]. Thus, not only spatial cell geometry [104], but also cell differentiation can be controlled by modifying the chemical structure of the hydrogel-based 3D support. This feature is important when handling iPSCs, which can easily undergo undesired phenotypes during in vitro culture. Noteworthy, synthetic polymers with a high acrylate content successfully combined with vitronectin to maintain pluripotency of iPSCs in vitro for a prolonged period of time [105]. Similarly, fully synthetic hydrogels based on Poly[2 -(methacryloyloxy)ethyldimethyl-(3-sulfopropyl) ammonium hydroxide] (PMEDSAH) were exploited to prevent spontaneous iPSC differentiation in vitro [106]. Several studies attribute this property to sulfonic groups, which mimic the function of the heparin sulphate present in proteoglycans, important extracellular components of iPSC culture systems [106]. On the other hand, hydrogels can also be opportunely modified to guide stem/progenitor cell differentiation towards specific phenotypes. A relatively simple inclusion in the hydrogel backbone of molecules derived from native ECM makes it possible to fabricate bioactive materials presenting well-defined biological cues. Recent studies [107] report the insertion of type I collagen to improve the differentiation of rat bone marrow MSCs towards hepatocyte-like cells using matrix-assisted 3D culture in vitro. Interestingly, Wang et al. [108] found that the simultaneous inclusion of fibronectin and type I collagen into a 3D system further improved the differentiation of human adipose-derived MSCs into functional hepatocytes. Hepatocytes showed ALB production and ammonia detoxification capabilities. Authors also reported a statistically significant increase in the expression of hepatocyte-specific genes including *ALB*, *HNF4A*, *AFP*, and *G6P* (glucose-6-phosphatase) on day 21 of differentiation in 3D conditions as compared to conventional 2D conditions.

The native ECM also provides mechanical support to the cells. Hydrogel-based materials can be finely tuned to induce a well-defined mechanical response in target cells. In vivo, the most prevalent mechanism involved in ECM-cell interaction is mediated by different classes of integrins expressed on the cell surface. Such adhesion molecules selectively interact with specific ECM components (e.g., fibronectin) inducing the formation of focal adhesion, which transfer external mechanical stimuli to the intracellular cytoskeleton [109]. These interactions induce biological responses that modulate cell behavior. Following this rationale, Wang et al. [110] established a 3D hydrogel scaffold based on polyethylene glycol (PEG) functionalized with fibronectin as ECM-like moieties, and cellularized it with human hepatoma-derived cells. Interestingly, the presence of fibronectin induced hepatocytes to produce ALB and to express higher levels of liver-specific genes.

Different mechanical cues (e.g., stiffness) can be generated by chemical modification of the polymeric backbone. Hwang et al. [111] proposed a micropatterned hydrogel matrix mainly composed of heparin. The chemical composition was accurately set to confer a soft consistence to the hydrogel (elastic modulus ~ 400 Pa). Then, the matrix was cellularized with human adipose-derived stem cells and cell differentiation towards hepatic phenotypes was evaluated in 3D conditions. Compared to a stiffer hydrogel matrix (elastic modulus ~ 43 kPa), this system significantly promoted stem cell maturation. Similar studies of pluripotent stem cell differentiation into hepatocyte-like cells were performed by other groups using hydrogels composed of unmodified [112] or collagen-coated [113] polyacrylamide. These studies concluded that a soft matrix facilitates the maturation of human iPSCs [112] and resident liver stem cells [113] toward the hepatic phenotype. This evidence is consistent with the observation that by mimicking the mechanical properties of hepatic parenchymal ECM (reported to be 0.6–1.2 kPa [114]), hydrogels help prolong the differentiated phenotype of primary hepatocytes in vitro [115].

In addition to the hydrogel-based 3D culture, the repopulation of decellularized tissues with specific cells represents a valid approach for the rebuilding of liver structures. This approach is highly effective thanks to the application of recently developed technologies, which allow for the maintenance of a high percentage of native ECM after the decellularization process [116]. However, one of the most limiting factors is related to the shortage of suitable donor tissues and organs. Moreover, and not less important, the geometry of decellularized organs is not modulable as hydrogel-based biomatrices. The use of decellularized extracellular matrix (dECM) emerged to overcome such weaknesses. dECM is commonly obtained following enzymatic digestion and lyophilization of decellularized tissues and organs and can be formulated as a highly concentrated solution, which can be easily handled and shaped into 3D matrices [117,118] using simple methods. Compared to hydrogels, dECMs are biochemically more complex, due to a high and undefined content in active substrates including a wide plethora of bioactive proteins, carbohydrates, and growth factors [119]. Among these substrates, fibronectin, type I and IV collagen, and proteoglycans are the most relevant for liver dECM, as they participate in cell attachment, survival and growth, and hepatic differentiation [120]. In accordance, Wang et al. [119] reported enhanced hepatic differentiation of iPSCs when these cells were cultured in a 3D liver-derived dECM rather than a 3D polymeric scaffold.

Of note, the composition of dECM largely depends on the tissue where it was extracted from. This means that each dECM might guide cell differentiation in a tissue-specific manner [121]. This was confirmed by Vyas et al. [122], who showed how liver-derived dECM could promote human fetal liver progenitor cell differentiation into mature hepatocytes with xenobiotic catabolic activity.

Despite dECMs showing promise in supporting and guiding stem/progenitor cell differentiation, they are not suitable for the generation of complex 3D structures due to their poor mechanical properties [123]. Low viscosity and poor resistance to shear stress drastically limit the processing of dECMs with advanced manufacturing technologies as 3D printing [124], 3D bioprinting [125] or electrospinning [126]. To address this issue, a widely-employed approach is to combine dECM with a hydrogel to obtain a hybrid bioactive material with better mechanical properties. Yu et al. [127] fabricated a micropatterned iPSC-laden 3D liver-like construct using a mixture of liver dECM and gelatin methacrylate (GelMA). The hydrogel conferred adequate mechanical properties to the liver dECM, enabling the processing of the combined material in a 3D bioprinter, and maintaining in parallel a high content in bioactive molecules. Interestingly, the matrix could guide cell organization and promote the maturation of iPSCs into hepatocyte-like cells in vitro.

Overall, the reviewed studies highlight the advantages of using 3D tissue models over conventional cell-based assays for creating functional tissues from primary or iPSC-derived cells that are realistic enough to be exploited for disease modeling and drug screening applications. As new biomaterials are discovered, and the mutual interactions between cellular and non-cellular components in a tissue are deciphered, new methods to grow functional tissues are expected to emerge. Challenges that remain to be addressed include a limited ability to control a system in vitro that is physiologically highly dynamic.

## 4. Concluding Remarks and Open Questions 

Because of its vital functions, the liver is susceptible to a wide range of diseases often requiring liver transplantation. Understanding cellular and molecular mechanisms of liver development, regeneration, and pathogenesis is therefore crucial for the design of alternative treatments to organ transplantation. In addition, the availability of an in vitro cell model able to properly and reliably recapitulate liver functions would reduce the need of animal experimentation in drug testing and development of new therapies. Owing to the presence of all enzymes of the xenobiotic pathway and, more generally, to a metabolic competence similar to the physiological one, primary human hepatocytes are considered the gold standard for both toxicological and pharmacological screening [128]. However, due to a scarcity of healthy donor liver tissue, obtaining these cells is challenging. Moreover, primary cultures cannot be expanded long-term and tend to dedifferentiate, thus they are unsuitable for large scale and long-term studies. Another important limitation of using primary cultures is the inter-donor variability [129]. Immortalized hepatocytes, normal and cancerous, are better expanded, but display an immature phenotype and might therefore result as unsuitable for many drug screening applications, which demand for native physiological functions to be adequately recapitulated in order to provide meaningful information [130,131]. An exception is the hepatic stem cell line HepaRG [132], which has emerged as the most appropriate surrogate to primary human hepatocytes and is considered a good alternative, even with limitations, for drug testing among the hepatoma cell lines [130,133]. Similar considerations apply to cholangiocytes [134], especially for those lining the IHBDs.

**Figure 1:**
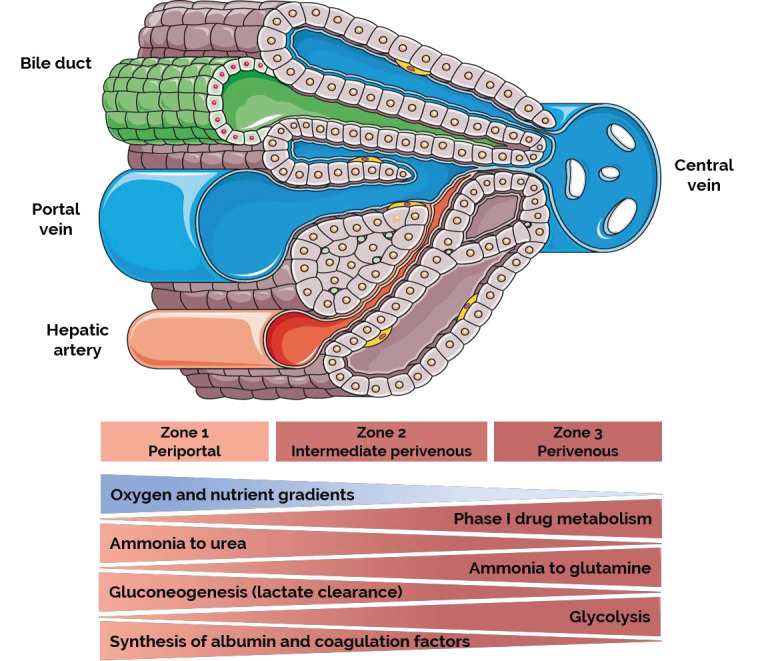
Liver metabolic zonation. Image adapted from Servier Medical Art (www.smart.servier.com).

The iPSC technology allows for the obtainment of an unlimited supply of cells from patients, including those with rare genetic disorders. iPSC-derived human hepatocytes represent a valuable tool for the rapid screening of a large number of compounds in vitro, and thus, show great promise for the identification of novel liver therapies. However, the reduced activity of phase I and II enzymes of the xenobiotic metabolism argues that iPSC-derived hepatocytes are not yet an ideal platform for preclinical pharmaceutical development [130]. Recent efforts to improve differentiation consider the unique liver microenvironment during embryonic development and organ homeostasis. Many drug responses in vivo are mediated by a complex interplay among several different cell types and most available in vitro models are unable to recapitulate heterotypic cell–cell interactions. The (re)building of a relevant liver in vitro model not only does require the incorporation of various cell types, including hepatocytes, cholangiocytes, LSECs, stellate cells, and Kupffer cells, but also requires the alignment of hepatocytes and cholangiocytes along the sinusoidal spaces and periportal tracts, respectively, and last but not least, the application of mechanical stimuli, and fluid flow. Blood flow is a central determinant for liver function. Blood flows from the periportal to the central vein and consists of a mixture of nutrient-rich but poorly oxygenated blood from the portal vein and highly oxygenated blood from the hepatic artery. Gradients of oxygen, nutrients, hormones, and metabolites along the periportal–central axis are created as the blood flows through the sinusoids, generating functional hepatocyte heterogeneity, a phenomenon referred to as ‘liver zonation’ [135]. Three zones can be distinguished (Figure 1). Periportal Zone 1, consists of 6–8 hepatocyte layers that receive blood enriched in oxygen and nutrients and control glycogen metabolism, amino acid utilization, and ammonia detoxification; intermediate perivenous Zone 2, consists of 6–10 hepatocyte layers with a major role in xenobiotic metabolism; and perivenous Zone 3, is formed by 2–3 hepatocyte layers that surround the central veins and perform biotransformation reactions, glutamine synthesis, and glycolysis [136,137].

This broad spatial and functional heterogeneity suggests that hepatocytes in different lobule zones might have distinct susceptibilities and tolerance to injury, as well as regenerative capabilities. Synthetic reconstruction of zonation in vitro is therefore required to adequately recapitulate responses of mature hepatocytes. Metabolic zonation appears to be primarily driven by factors secreted by endothelial cells of the hepatic central vein, including members of the Wnt/β-catenin pathway. Three signal modulators secreted from hepatocytes or cholangiocytes, namely WNT7B, WNT8B, and WNT inhibitor factor 1 (WIF-1) were identified by Mitani et al. as determinants of zone-specific hepatic properties in ESC/iPSC-derived hepatocyte-like cells [138]. Physiologically, Zone 1 hepatocytes exist near the cholangiocytes, while zone 3 hepatocytes are physically distant from the cholangiocytes. Accordingly, the authors obtained zone 1 hepatocytes with enhanced urea production and gluconeogenesis capabilities by simply exposing induced hepatocytes to conditioned medium from cholangiocytes. On the other hand, when induced hepatocytes were cultured with conditioned medium from hepatocytes, cells achieved zone 3-specific hepatic properties, including enhanced glutamine production and CYP1A2 metabolism.

While the role of the multicellular components has been increasingly highlighted, only a couple of reports have described the concomitant differentiation of hepatic and biliary lineage in a co-culture system [79,91,139]. Hepatic organoids consisting of both hepatocytes and cholangiocytes showed a functional bile canaliculi network, and could be used to model drug-induced cholestasis, as well as loss of bile canaliculi network and ductular reaction [79]. Multicellular organoids undoubtedly represent a significant advance toward the production of a surrogate hepatobiliary tissue, however, most of these systems retain a fetal-like state even in prolonged culture. Developmental studies have shown that the factors inducing hepatic or biliary fates are often opposing in nature, making the de novo assembly of liver structures endowed with a biliary system very challenging. Moreover, the specific identity of cells generated with current protocols remains undefined. Not only the liver, but also the mature biliary epithelium displays a phenotypic and functional heterogeneity. At least two different types of cholangiocytes can be distinguished. Small cholangiocytes populate the periphery of the biliary tree, while large cholangiocytes populate larger diameter IHBDs and EHBDs. Small cholangiocytes are primitive, undifferentiated cells that do not display secretory activity, while large cholangiocytes are highly specialized cells that secrete and modify the bile [140,141]. Induced cholangiocytes obtained by Dianat et al. [49] are compatible with small cholangiocytes, while those obtained by Sampaziotis et al. [64] are functionally compatible with large cholangiocytes, even maintaining intrahepatic cholangiocytes characteristics [142]. In both cases, the cells show a fetal phenotype, corresponding more precisely to a stage between fetal and fully mature bile ducts. Thus, as for induced hepatocytes, these systems require further improvement. 

The creation of surrogate tissues in vitro can leverage methods from tissue engineering and microfluidics, which can help achieve biological, chemical, and mechanical cues typical of the physiological environment. Biomaterials can be precisely manufactured in order to confer peculiar characteristics to guide differentiation of iPSCs toward a certain cell type. This strategy has been exploited to create cellular constructs with the potential to regenerate tissues and organs, such as tendons, nerves, and liver [143], and also to create platforms for disease modeling and drug testing [144]. On the other hand, microfluidic represents a valid tool for the study of iPSC behavior and differentiation [145]. Microfluidic technology allows the creation of high-content drug screening systems, which better simulate tissue morphology at a micron scale [146]. Interestingly, hepatic zonation can be obtained exploiting chip technology [147], making it possible to study the physiology and pathology of periportal and perivenous hepatocytes, and opening up new paths for disease modeling and more tailored drug testing.

In view of the above, dissecting the heterogeneous cellular composition of the hepatobiliary system (cell-type heterogeneity and cell–cell heterogeneity) and the complex interplay of cell–cell and cell–matrix interactions in homeostatic and pathological conditions is crucial to recreate tissue surrogates for disease modeling, validation of existing therapeutics, and screening of novel agents. Single-cell transcriptomics are opening up the possibility that new signaling circuits and new subpopulations of cells can be found, which will likely translate, for a given cell type, in the design, in the near future, of distinct culture protocols depending on what requirement is to be satisfied. Indeed, in an era in which a lot of effort is being put into developing standardized differentiation protocols, one protocol might not fit all. Finally, advances in genome editing are greatly improving the modeling of a variety of human disorders. However, strategies for minimizing off-target mutations, as well as methods for detection of unintended off-target mutations, are strongly needed to encourage the use of such technologies in research and clinical applications.

## Figures and Tables

**Table 1:** List of small molecules used for hepatobiliary cell fate specification.

Differentiation Stage	Small Molecule for Induction	Pathway Involved	Effect
DE	Activin A	TGF-β	Activation
	LY29002	IRS/PI3K	Activation
	CHIR99021	Wnt/β-catenin	Activation
Hepatoblast	A83-01	TGF-β	Inhibition
	CHIR99021	Wnt/β-catenin	Inhibition
	Dihexa	HGF/Met	Activation
	Foskolin	PKA	Activation
	SAG	Sonic Hedgehog	Activation
	Vitamin C	Non-specific	Modulation of cell senescence
Hepatocyte-like cells	DMSO	Non-specific	Increase in cell differentiation
	DEX	Glucocorticoid	Activation
	Dihexa	HGF/Met	Activation
	A83-01	TGF-β	Inhibition
	Sodium butyrate	p21Waf1/Cip1	Activation (cell cycle arrest)
Cholangiocyte progenitors	Activin A	TGF-β	Activation
	Retinoic acid	Wnt/β-catenin	Inhibition of canonical Wnt and activation of non-canonical Wnt pathway
Cholangiocyte-like cells	Activin A	TGF-β	Activation
	Retinoic acid	Wnt/β-catenin	Inhibition of canonical Wnt and activation of non-canonical Wnt pathway
	DEX	Glucocorticoid	Activation
	Sodium pyruvate	Energy metabolism	Increase in cell survival
